# Treatment of Lower Back Pain—The Gap between Guideline-Based Treatment and Medical Care Reality

**DOI:** 10.3390/healthcare4030044

**Published:** 2016-07-15

**Authors:** Andreas Werber, Marcus Schiltenwolf

**Affiliations:** 1 Department of Orthopedics and Orthopedic Surgery, University Hospital Giessen, Klinikstr. 33, 35392 Giessen, Germany; andreas.werber@ortho.med.uni-giessen.de; 2 Department of Orthopedics and Traumatology, University Hospital Heidelberg, Schlierbacher Landstr. 200a, 69118 Heidelberg, Germany

**Keywords:** low back pain, guideline-based treatment, somatisation

## Abstract

Despite the fact that unspecific low back pain is of important impact in general health care, this pain condition is often treated insufficiently. Poor efficiency has led to the necessity of guidelines addressing evidence-based strategies for treatment of lower back pain (LBP). We present some statements of the German medical care reality. Self-responsible action of the patient should be supported while invasive methods in particular should be avoided due to lacking evidence in outcome efficiency. However, it has to be stated that no effective implementation strategy has been established yet. Especially, studies on the economic impact of different implementation strategies are lacking. A lack of awareness of common available guidelines and an uneven distribution of existing knowledge throughout the population can be stated: persons with higher risk suffering from LBP by higher professional demands and lower educational level are not skilled in advised management of LBP. Both diagnostic imaging and invasive treatment methods increased dramatically leading to increased costs and doctor workload without being associated with improved patient functioning, severity of pain or overall health status due to the absence of a functioning primary care gate keeping system for patient selection. Opioids are prescribed on a grand scale and over a long period. Moreover, opioid prescription is not indicated properly, when predominantly persons with psychological distress like somatoform disorders are treated with opioids.

## 1. Introduction

The onset of chronic lower back pain (cLBP) is based upon various factors. A former history of LBP is the most consistent risk factor for transition from a baseline of a pain-free state [1]. LBP is marked as chronic if the pain occurs on more than half of the days of the last half-year. In a broader sense, cLBP is defined as the final point of a chronification process including the following characteristics: generalization of pain, changing areas of pain, other complaints that cannot be explained merely somatically (buzzing in one’s ears, digestive disorders, insomnia). Furthermore, changes in behaviour are concomitant, for instance increasing consumption of medication, alternating presentation of different symptoms, avoidance of exercises and social withdrawal [2].

The risk of suffering from LBP differs significantly within the general population. Especially psychological distress in terms of dysfunctional behaviour plays a decisive role in the development process [2]. In less than 10% of the cases LBP can solely be explained somatically. In fact, LBP is often an alternative expression of physical stress symptoms of which the patients are seldom aware. LBP is rather a medical condition than a complete medical entity. In combination with other symptoms like depression or anxiety disorders, cLBP is an expression of distress [3].

Several countries developed guidelines in order to provide a systematic approach for treatment of cLBP with similar procedures both for diagnosis and treatment [4,5]. However, both patients and physicians are seldom aware of how to deal properly with LBP according to recommendations of common available guidelines [5]. Monomodal therapy often leads to insufficient therapeutic response, hence it is important to identify the distinct factors of causing pain and treat them properly in terms of a multidisciplinary (=multimodal) therapeutic approach [6].

The aim of this article is to outline the medical care reality in Germany in terms of diagnosis and treatment of LBP by presenting processed statistical data of German insitute for tarification system for hospital care. Despite increasing numbers of diagnostic and therapeutical procedures, the effectiveness of treatment is still poor. Lacking awareness of common available guidelines of how to cope with LBP leads both to an increasing dependency of the patient from the therapist due to insufficient perception of self-effectiveness of a therapy and furthermore to outdated treatment approaches heavily influenced by the habits of the therapist.

### 1.1. Epidemiology

Life time prevalence for acute LBP (aLBP) varies between 58% and 85% (in Western industrial nations), one-year prevalence varies between 20% and 40% and point prevalence for Germany is 8% for men and 14% for women. The biggest incidence is found in the fourth decade of life [7,8]. The resulting costs (both direct costs for medical treatment and indirect costs for stoppage and/or retirement pay) varies between €400 and €7000 per patient and year. In total, costs of over 50 Billion Euros occur solely in Germany each year. Six percent of all direct costs for medical treatment, 15% of all incapacity for work and 18% of all early retirements are associated with LBP [8]. Education and appearance of LBP correlate significantly [5]. A representative study of how to coop with available guidelines proved that higher education is a protecting factor for suffering from LBP. A total of 82.9% of the participants with lower education levels suffered from lower back pain at least once in their lifetime compared to only 62.4% of people with university degrees. Especially women with lower education had a significantly higher risk of suffering from LBP. In contrast to this, participants with an university-entrance degree had a 70% lower risk, those with completed academic studies had a 60% lower risk of developing LBP [5].

### 1.2. Data Source

Our data analysis was based upon free availabe health care data provided by the German insitute for tarification system for hospital care—InEK (http://www.g-drg.de). To ensure compensation for general hospital services in Germany, a consistent performance-oriented remuneration system was established according to the Hospital Finance Act (KHG). Basis for this is the G-DRG-system (German-Diagnosis Related Groups-System), in which every treatment case is compensated by flat-rate payment depending upon the according DRG. Duties associated with the implementation, further development and maintenace of the payment system were assigned by the German Hospital Federation and health insurance associations to the InEK GmbH as the German DRG institue. All provided in-patient and out-patient data was analysed and classified in terms of procedures and body region to allow statements of common diagnosis and treatment procedures.

## 2. Diagnostics

According to the German insitute for tarification system for hospital care—InEK—the number of MRIs of the lumbar spine rose from 40,000 in 2004 to more than 75,000 in 2007 and to more than 385,000 in 2015 (inpatient treatment). An analysis of six random trials with a total of 1084 patients showed that with diagnostic assessment neither in short-term nor in medium-term improved clinical outcome could be achieved, provided the fact that there was no evidence for serious underlying conditions, so-called “red flags” (Table 1) [9].

Psychosocial risk factors, so-called “yellow flags”, gained importance as predictors of chronification and extent of subjective impairment (Table 2). The underlying tendency for chronification occurs on several levels: typical somatic factors like heavy labor are eclipsed by a converse behavior as a result of a sedentary lifestyle leading to a degradation of the musculoscelettal system, influenced by psychosocial risk factors like depressiveness and acquired helplessness in terms of coping strategies [10].

## 3. Therapy

### 3.1. Monomodal Therapy

Subjective impairment with inability to participate in terms of activities of daily-living due to LBP lead to physician consultation. Since chronification factors from psychosocial co-morbidity are by definition not relevant for aLBP, the usual therapy approach is merely a medical treatment with analgesia [11]. Another aspect is a physician’s recommendation of avoidance of any physical strain despite the fact being counterproductive in terms of a chronification process. Actually, bed rest should be avoided with LBP, except for ischialgia where no distinct recommendation is stated; however, an active lifestyle should be advised [12].

#### 3.1.1. Medical Therapy

According to the pain ladder of the WHO, NSAID medication is used for mild pain. For moderate to severe pain, mild and strong opioids are usually prescribed. Co-Analgesia like antidepressants and antiepileptic drugs augment the analgesic effect of the basic medication. An Australian study showed that for aLBP neither diclofenac nor spinal manipulative therapy appreciably reduced the number of days until recovery compared with placebo drug or placebo manipulative therapy [11]. During the transition from acute to chronic LBP it is pretty common to escalate pain medication from NSAIDs to opioid medication. Both in the USA and in Germany, the number of prescriptions of opioids increased by more than 100% between 1997 and 2004 [13]. However, in the long term, no difference in pain relive can be stated for NSAIDs or opioids. Hence, an interdisciplinary guideline for long-term opioid application for nun-tumor pain was published in Germany in 2010 (LONTS) [14] and revised in 2015 [15]. Continuous application of opioid medication is inconsistent with the strict indication considering contraindications of the guideline. For instance, an application of opioids for more than 12 weeks is only recommended if an essential and comprehensible pain relief is achieved without dosage escalation. Patients with intermittent pain episodes (for instance trigeminus neuralgia), continuous headaches with physical not sufficiently explainable symptoms as well as patient's with depressive or anxiety disorders should not be treated with opioids. Continuously decreasing analgesic effects result in a paradox hyperalgesia and cognitive impairment, thus leading to abusive intake of opioids. An evaluation of health insurance data of a German statutory health insurance company between 2006 and 2010 showed that the number of prescriptions of opioid prescriptions increased virtually linearly. Prescriptions of mild opioids were decreasing for non-tumor pain, but increasing for tumor pain, while the number of prescriptions of strong opioids was increasing both for tumor and non-tumor pain. Differences occurred in terms of duration and kind of the preferred substances, including the considerations of common contraindications (e.g., somatoform disorders). The majority of strong opioids being prescribed for non-tumor pain were fentanyl pain patches for 40 to 45 year old males with average annual costs of 1833 Euros per patient. Out of 21,000 patients with somatoform pain disorder, 44.4% were treated with opioids (20.7% with mild, 23.7% with strong opioids). Prescribing behavior was often not consistent with common indications and contraindications.

#### 3.1.2. Interventional Therapy

During the last few years, a massive increase of spinal injections in the lumbar region from 778,362 in 2006 to 1,197,302 in 2009 (inpatient and outpatient treatment) was observed, a trend similar both in the USA and in Germany [16]. Effects of injections can be stated, but only last for a limited period of time [17]. Beside the doubtful effectiveness of those therapies, the risk of side-effects like infection and/or vascular lesions are considerably increased leqading to problematic cost-benefit-ratio [18].

#### 3.1.3. Surgical Therapy

In Germany, the number of lumbar spinal surgery increased from 165,000 in 2006 to 250,000 in 2009 an to more than 705,000 in 2015 (inpatient treatment), comparable to the development in the USA (Table 3). Although leading to considerable costs and complications by an increasing number of spinal surgeries, a verifiable benefit of quality of life cannot bet stated [18]. Several studies showed that less than half of the patients with spinal surgery became pain free, independent from the applied surgical technique [9,17,18].

#### 3.1.4. Synopsis of Monomodal Therapy

Considering present studies, monomodal strategies—conservative, interventional or operative—show only little effects in terms of treatment of cLBP. In a recent meta-analysis with a total of 76 trials reporting on 34 treatments it was observed that only 50% of the investigated treatments had statistically significant effects and for most the effects were small or moderate [19]. This meta-analysis revealed that the analgesic effects of many treatments for non-specific low back pain are small and that they do not differ in populations with acute or chronic symptoms. A main reason for this outcome is that cLBP has a multifactorial pathogenesis which is not covered sufficiently with a monomodal therapy approach. Pointless therapeutical action (due to unfulfilled patient’s desires and physician’s increasing readiness to act) leads to a medicalization of a common pain phenomenon, ignores patient’s own resources and replaces theses resources by ongoing escalation of therapeutical approaches. Since the patient’s resources are hereby ignored paternalistically, a sustainability of a therapeutical approach is not reachable [2,5,13].

### 3.2. Multidisciplinary Therapy

This is understood as the application of different kinds of body therapy (exercises for strength, endurance and mobility as well as body perception) and coequally psychotherapy (cognitive behavioural therapy or psychodynamic therapy) in the presence of chronified pain syndromes. The objective is an active somatic and psychological therapeutical approach. Passive methods should be avoided. All therapies should be in a group setting with a maximum of 8 participants, seldom as an individual therapy. The key aspect of a multidisciplinary therapy is the collaboration of all involved persons under an individual dysfunctional concept of each patient. At least once a week, the patient’s individual progress and drawback is discussed in a team and the patient’s treatment focus is adjusted accordingly. It could be shown that only intensive (more than 100 h) multidisciplinary biopsychosocial rehabilitation with functional restoration reduces pain and improves function in patients with CLBP chronic low back pain, while less intensive interventions did not show improvements in clinically relevant outcomes [20].

#### 3.2.1. National Disease Management Guideline Recommendations

National disease management guidelines (NVL) incorporate medical guiding decisions and criteria regarding diagnosis, management, and treatment of chronic diseases based upon systematic developed and evaluated therapy strategies. In Germany, the German Agency for Quality in Medicine (ÄZQ) coordinates a national program for disease management guidelines, similar to the National Guideline Clearinghouse in the USA [5].

In terms of acute lower back pain, extensive diagnostic and therapeutical interventions should be avoided and the mainly harmless natural history of the condition should be monitored [21]. However, it is important that distinct warning signs, so-called “red flags”, of serious physical illness are excluded reliably and at an early stage. In case of a red flag, a target-oriented therapy should be pursued including appropriate diagnostic and therapeutical instruments, for example MRI and laboratory diagnostics. After the exclusion of red flags, imaging diagnostic should be avoided completely for at least 4 weeks since the information yield is negligible. It is important to explain this approach to the patient, taking his complaints seriously. In case of persisting pain (pain reduction less than 50% after 4 weeks), a reevaluation of the clinical findings should be carried out, including imaging diagnostics, preferably MRI. A short-term administration of medicatio—without recommendation of a distinct substance—can be considered, even if a relevant benefit is not verifiable [22]. In fact, active exercises under the patient’s own authority should be encouraged while passive therapies (including physiotherapy) should be avoided. Long-term certificates of incapacity should not be prescribed since with the continuation of inability to work the demands of the working place increase as as the demands of the labor market in case of job loss. With increasing inability to work and the loss of physical and psychological strength, the probability of occupational reintegration decreases accordingly.

Due to psychosocial risk factors, so-called “yellow flags”, of chronification of LBP, a merely somatically based therapeutical approach is to be considered only partially promising. According to current studies, the cost-benefit-ratio of surgical approaches (for instance disc prostheses or mono-segmental fusions) is inconsistent, hence being not recommended [16,23]. A multidisciplinary therapy including psychotherapy for treating CLBP is not just equal to a surgical approach in terms of pain reduction but also more cost-effective and less risky. Multidisciplinary pain therapy is the central therapy recommendation of the NVL encapsulating a combination of medical and psychotherapeutical methods as well as movement therapy addressing a patient’s individual dysfunctional concept [24]. The patient’s own capabilities should be encouraged while fears and conflicts should be overcome to achieve greatest possible self-effectiveness. One mandatory requirement for a therapy’s sustainability is the movativation of the patient for self-responsible acting. The degree of chronification as a substantial prognostic factor includes a generalization of pain areas from local to widely spread pain and other complaints that cannot be explained merely somatically (buzzing in one’s ears, digestive disorders, insomnia). The effects of a multidisciplinary pain therapy are inversely proportional to the number of pain areas [25]. With persisting pain after 12 weeks of proper treatment (according to common guidelines) and relevant activity limitations, a multidisciplinary therapy should be evaluated. In the presence of psychological risk factors, a multidisciplinary therapy should be considered after 6 weeks of unsuccessful treatment.

#### 3.2.2. Synopsis of Multidisciplinary Therapy

The treatment of LBP is characterized by a large number of acute medical oriented methods, for instance diagnostic imaging, injection therapy or chiropractic therapy. Due to only little additional information yield, diagnostic imaging should be reduced to a minimum, hence treatment is usually not affected.

Psychosocial risk factors of chronification are only respected sparsely. In particular, the number of interventional procedures increased significantly with limited evidence for efficiency as well as increased costs for treatment and increased risk of complications in the healing process.

**Figure 1:**
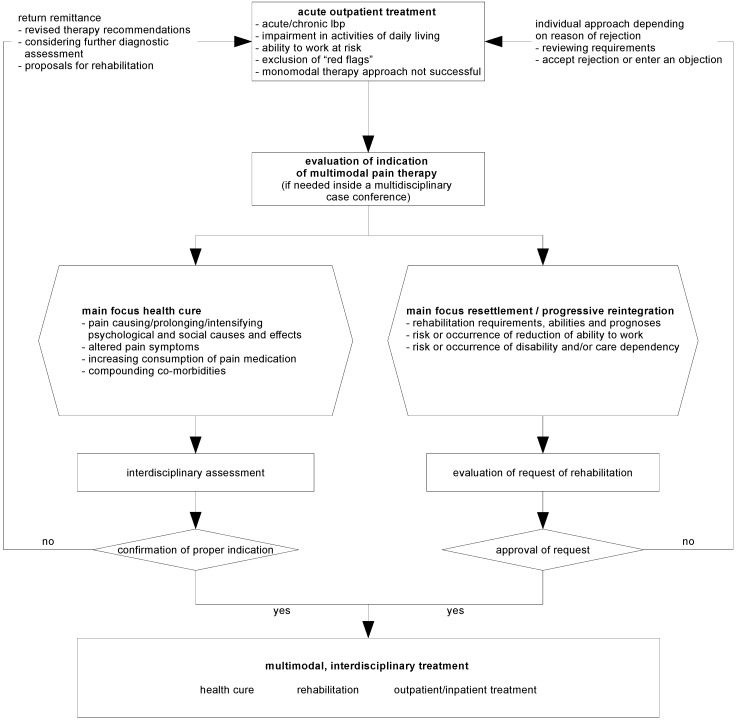
Algorithm for treatment of patients with unspecific LBP according to the suggestions of the National Disease Management Guidelines (NVL).

Based on the recommendations of the national disease management guideline for LBP, four aspects of treatment should be taken into account:

1. exclusion of specific causes of the complaints, red flags, by considering the medical history and a thorough clinical and neurological examination.
2. identification of psychosocial risk factors, yellow flags, and adjusting the treatment accordingly including psychological assistance.
3. workflow for patients with unspecific lower back pain according to the suggestions of the National Disease Management Guidelines (NVL) (Figure 1).
4. interdisciplinary diagnosis: Due to the increasing relevance of psychosocial risk factors in case of persisting pain, a transition to chronic pain syndromes has to be expected. Hence, further treatment of subacute or even chronic pain syndromes must be accompanied by an interdisciplinary diagnostic approach. In case of persisting pain after 12 weeks (or 6 weeks with psychosocial risk factors), both re-evaluation with regard to specific causes of the pain syndromes and diagnostic imaging (preferably by MRI) should be carried out. If no specific cause can be verified, a chronification of the LBP is very likely. Therefore, an early activating and self-effective therapy is of high significance. Interventional and surgical procedures, especially in terms of cost-benefit ratio, are negligible. With larger distance to re-integration to work and daily life, the patient’s morbid gain will increase, therefore the patient’s motivation will be presumably be impaired. Furthermore, the outcome of the therapy will be affected as well, hence it is of great significance to include the patient into self-effective therapeutical possibilities.

## 4. Discussion

The increasing number of diagnostic and therapeutical procedures (medical imaging, interventional and surgical procedures) with no verification of effectiveness and high expectations of the patients for successful treatment using technical solutions demonstrate that LBP is not merely a medical, but rather a social phaenomenon. Despite lacking evidence, both patients and service providers agree that a massive medicalisation of the phenomenon back pain is indispensable. However, this view leads to a further passiveness of the patient in terms of self-effectiveness of any therapeutical approach and an increasing level of dependence of the patient from the therapist.

A lack of awareness of common available guidelines how to cope with LBP can be stated with an uneven distribution of existing knowledge throughout the population. Passive coping strategies like taking pain medication or ointment therapy are favored over active coping strategies like gymnastics, physical activities, and relaxation exercises. Respondents with a higher level of education suffer significantly less often from LBP and tend toward active treatment strategies. Respondents with lower levels of education more often demand passive treatment strategies. The general population, especially those with lower education, is not sufficiently aware of behavioral strategies for managing LBP, as proposed in available guidelines.

The option of an active self-effective acting is ignored. The national medical guidelines for unspecific LBP establish a therapeutical concept at different stages offering evidence-based recommendations. Small effects of common treatment approaches for LBP yielded to the necessity of interdisciplinary created, generally accepted and evidence-based recommendations for treatment.

However, one major problem is the focus of the guideline on unspecific LBP, because every monomodal therapeutical approach (for instance interventional and surgical procedures) can be legitimized by arguing that a specific cause for the pain syndrome is present. Because of the insufficient distinctiveness between specific and unspecific pain syndromes, it will be possible to skip the recommendations of the guideline by referring to a specific cause devaluing the concept of a multidisciplinary therapeutical approach.

## 5. Conclusions

Knowledge of treatment guidelines for LBP is not sufficiently available in the general population. Physicians should address the knowledge of patients about the rightful treatment behavior and should provide guideline-oriented treatment strategies. Active rules of management should be emphasized while the importance of passive rules of management should be downgraded.

## Figures and Tables

**Table 1:** “Red Flags”—specific causes of back pain symptoms.

Anamnesis	Specific Cause
history of fall and/or accident	fracture
drug abuse	spondylodiscitis
malignant primary disease	metastasis, pathological fracture
immunosupression (e.g., AIDS)	spondylodiscitis
chronic infection	spondylodiscitis
long-term cortisone intake	cortisone-induced osteoporosis
involuntary urination and defecation	conus-cauda-syndrome
paresis	nerve root compression

**Table 2:** “Yellow Flags”—risk factors of chronification of back pain symptoms.

Risk Factor
low work satisfaction
low social status
stress
age
female sex
possibility of morbid gain
passive lifestyle
nicotine, alcohol, drug abuse
obesity
insufficient self-regulation
little physical and psychological resources

**Table 3:** Number of procedures of inpatient treatment between 2014 and 2015 according to the German Procedure Classification System (OPS) ^1^.

OPS Code	Procedure Definition	Number of Cases
3-802	MRI of the vertebral column and spinal cord	280,631
3-823	MRI of the vertebral column and spinal cord with application of contrast media	92,779
3-841	MRT myelography	12,418
		385,828
5-83 (5.836)	surgical procedures of the spine (spondylodesis)	706,666 (64,812)
8-020.7	therapeutical injections of the intervertebral disc	9860
8-914	injections of nerve roots and injections near the spine for pain therapy	127,678
8-915	injections of peripheral nerves for pain therapy	136,700
8-916	injections of the sympathetic nervous system for pain therapy	2592
8-917	injections of vertebral joints for pain therapy	4452
		281,282

^1^ The German procedure classification (Operationen- und Prozedurenschlüssel-OPS) is the official classification for the encoding of operations, procedures and general medical measures in the inpatient sector and for surgical procedures in the outpatient sector. The German Institute of Medical Documentation and Information (DIMDI) publishes the OPS classification on behalf of the Federal Ministry of Health. Its use in inpatient care is laid down in § 301 Volume V of the German Social Security Code (SGB V) and for surgical procedures in the outpatient sector in § 295 SGB V (www.dimdi.de).
